# Pubic and Multiple Pelvic Fractures in Trauma Patients Are Associated With Intensive Care Unit Admission

**DOI:** 10.7759/cureus.95415

**Published:** 2025-10-25

**Authors:** Yuto Aramaki, Mizuki Mori, Kazunori Fukushima, Kiyohiro Oshima

**Affiliations:** 1 Department of Emergency Medicine, Gunma University Graduate School of Medicine, Maebashi, JPN

**Keywords:** injury severity scores (iss), intensive care unit, pelvic bone fracture, pubic bone, trauma

## Abstract

Introduction: Pelvic fractures usually result from high-energy trauma, often presenting as polytrauma and frequently requiring intensive care management. Anatomical severity is generally considered to be associated with intensive care unit (ICU) admission in trauma patients. This study investigated the specific types of pelvic fractures that are associated with ICU admission.

Methods: We retrospectively analyzed trauma patients transported to Gunma University Hospital in Maebashi, Japan, between April 1, 2017, and March 31, 2022. Pelvic fracture sites were categorized into 10 regions (left/right ilium, ischium, pubis, sacrum, and acetabulum). The relationship between fracture patterns and ICU admission was then analyzed.

Results: A total of 59 patients were included in the analysis. The Mann-Whitney U test and Fisher's exact test were used to analyze the relationship between the number of fractures, fracture type, and ICU admission. While there were no significant differences regarding unstable pelvic fractures (p = 0.06), patients with multiple fractures and pubic fractures were significantly more likely to be admitted to the ICU. Furthermore, patients with pubic fractures frequently had multiple fractures (p < 0.001), were more likely to show extravasation on imaging (p = 0.003), and require blood transfusion (p < 0.001) and endovascular treatment (p = 0.015) more often. Logistic regression analysis revealed that pubic fracture independently predicted ICU admission compared to other single fractures (odds ratio (OR) 11.9, p = 0.012).

Conclusion: Patients with multiple pelvic fractures and/or pubic fractures are more likely to be admitted to the ICU. These findings may assist in early triage decision for pelvic trauma patients.

## Introduction

Pelvic fractures are associated with high-energy trauma and frequently involve concomitant injuries to other body regions [1]. The mortality rate for pelvic fracture patients presenting with shock in the emergency department is reported to be as high as 40-50% [2-4].

Patients with severe conditions, such as those requiring mechanical ventilation, continuous medication, or massive transfusions, are managed in the intensive care unit (ICU). While pelvic fracture patients are prone to severe complications when they present with organ damage or vascular injuries [5-7], the specific impact of different fracture patterns on ICU admission has not been thoroughly investigated.

For trauma patients, anatomical scoring systems like the Injury Severity Score (ISS) [8] and New Injury Severity Score (NISS) [9] are considered to be superior to physiological indicators such as the Revised Trauma Score (RTS) [10] or the Simplified Acute Physiology Scale II (SAPS II) [11] for identifying individuals who require intensive care [12-14]. This led us to hypothesize that anatomical features of pelvic fractures may be associated with ICU admission. This study focused on the anatomical characteristics of pelvic fractures to determine which fracture patterns pose a risk for ICU admission.

## Materials and methods

This retrospective observational study was conducted at the Emergency Department of Gunma University Hospital in Maebashi, Japan. The study received approval from the Clinical Ethics Committee of Gunma University Hospital (IRB No. 2023-045). Patient consent was obtained via an opt-out method that was publicly announced on the hospital's Clinical Ethics Committee website.

Gunma University Hospital is a tertiary emergency hospital that annually admits an estimated 9,000 patients, including 4,500 individuals transported by ambulance. Patients diagnosed with pelvic fractures after CT examination for trauma between April 1, 2017, and March 31, 2022, were included.

Exclusion criteria were as follows: patients with pre-existing medical conditions leading to injury, patients transferred from other hospitals, walk-in patients, patients transferred to other facilities without hospital admission, patients transported in cardiopulmonary arrest (CPA), and patients who refused study participation. Imaging results were interpreted by two emergency physicians, with additional reference to reports from radiology specialists. Discrepancies in image interpretation were resolved by discussion.

The following information was collected from medical records: age, sex, mechanism of injury, vital signs at Emergency Medical Services contact, vital signs on arrival, past medical history, imaging findings, blood transfusions, treatments, hospital admission, ICU admission, Abbreviated Injury Scale (AIS) [15] for each injured region, ISS, and trauma-related mortality.

Mechanisms of injury were categorized as follows: occupant in a motor vehicle accident (MVA), pedestrian struck by a vehicle, fall from height, minor fall, motorcycle accident, and others.

Vital signs on arrival were used to calculate the RTS. The AIS and ISS were used to assess trauma severity. For this study, the pelvic bone was divided into 10 regions: left/right ilium, acetabulum, pubis, ischium, and sacrum. This classification was newly defined by us for the purpose of this study. Multiple fractures within the same region were counted as one. Sacral fractures extending across both sides were counted based on the longer fracture line. Pubic diastasis or sacroiliac joint disruptions without associated fractures were not counted. Organ injuries were determined based on AIS codes, with abdominal organs categorized as liver, kidney, spleen, bowel, and bladder. Mortality was defined as death within 30 days.

Statistical analysis

Continuous variables were presented as medians (with interquartile ranges), and categorical variables were shown as absolute (n) and relative (%) values. Patient information was categorized based on the ICU admission status. The Mann-Whitney U test was performed for continuous variables that were not normally distributed, and Fisher's exact test was used for binary variables. Subsequently, fracture types that were highly associated with ICU admission were analyzed. The analysis was conducted using logistic regression analysis and the receiver operating characteristic curve analysis. The variance inflation factor (VIF) was used to diagnose multicollinearity in the logistic regression analysis, with a VIF exceeding 10 defined as strong multicollinearity. Explanatory variables for the logistic regression analysis were selected based on factors associated with ICU admission suggested in previous literature, as well as age and sex.

All statistical analyses were performed with EZR (Saitama Medical Center, Jichi Medical University, Saitama, Japan), which is a graphical user interface for R (The R Foundation for Statistical Computing, Vienna, Austria). Odds ratios (ORs) were presented with 95% confidence intervals (CIs), and a p-value <0.05 was considered to be statistically significant.

Definitions

Shock is defined as a systolic blood pressure of less than or equal to 90 mmHg. Severe trauma is classified as having an ISS of 15 or greater [16, 17]. Low-energy trauma refers to a minor fall. The elderly are defined as individuals over the age of 60. An organ injury, such as to the brain or lungs, is an Abbreviated Injury Scale (AIS) score greater than 2. A patient has received a transfusion if they have had any blood transfusion within 24 hours of admission. A massive transfusion is the transfusion of more than 10 units of red blood cells.

## Results

**Figure 1:**
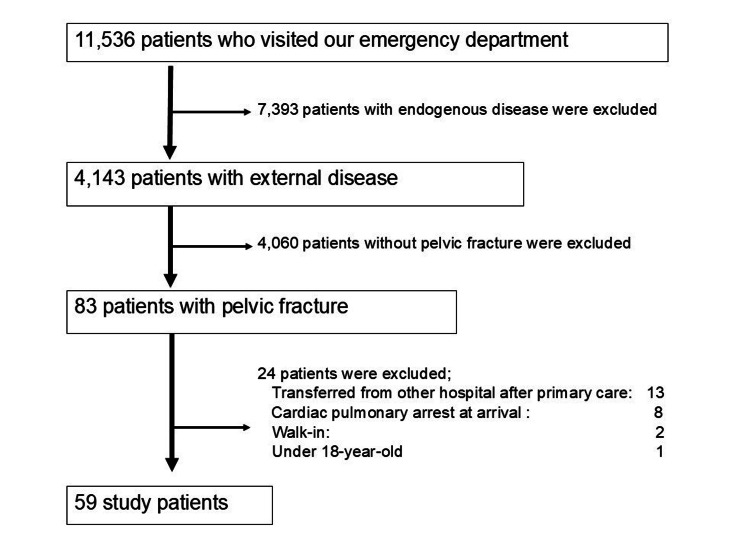
Flow diagram of the study population.

During the study period, 11,536 patients were transported to our hospital, with 4,143 cases that were attributed to blunt trauma. Of these, 83 cases were diagnosed with pelvic fractures, and 59 were included in the analysis (eight excluded due to CPA on arrival, 13 excluded due to referral/transfer, two walk-ins, and one pediatric case) (Figure 1).

The patient characteristics are presented in Table 1.

**Table 1:** Clinical characteristics of the patients (N = 59). AO/OTA: Association for Osteosynthesis Foundation and Orthopedic Trauma Association

Clinical characteristics	
Age (y/o)	69 (51.5, 79.5)
Male	30 (50.1%)
Injury mechanism	
Low-energy	15 (25.4%)
High-energy	44 (74.6%)
All death	1 (1.7%)
Abbreviated Injury Scale (AIS) of the pelvis	2 (2, 2)
Injury Severity Score (ISS)	10 (4, 16.5)
Fracture region (including duplications)	
Ischium	34 (57.6%)
Pubis	33 (55.9%)
Ilium	26 (44.1%)
Sacrum	17 (28.8%)
Acetabulum	18 (30.5%)
Single region fracture	16 (27.1%)
Multiple region fractures	43 (72.9%)
Bilateral	6 (10.2%)
Ipsilateral	30 (50.8%)
Organ damage (AIS ≥ 3) (including duplications)	19 (32.2%)
Lung	13 (22.0%)
Brain	8 (13.6%)
Intestinal tract/mesentery	2 (3.4%)
Kidney	3 (5.1%)
Liver	1 (1.7%)
Genitals	2 (3.4%)
Spleen	2 (3.4%)
Heart	0 (0%)
Bladder	1 (1.7%)
AO/OTA fracture classification	
A1	15 (25.4%)
A2	32 (54.2%)
A3	2 (3.4%)
B1	4 (6.8%)
B2	3 (5.1%)
B3	0 (0%)
C1	1 (1.7%)
C2	1 (1.7%)
C3	1 (1.7%)

The mechanisms of injury were as follows: nine cases involving an occupant in an MVA, 18 cases involving a pedestrian struck by a vehicle, 13 cases involving a fall from height, 15 cases involving a minor fall, three cases involving a motorcycle accident, and one other case (caught in an avalanche).

Table 2 summarizes the patient characteristics based on the ICU admission status.

**Table 2:** Comparison between the non-ICU-admitted {ICU(-)} and ICU-admitted {ICU(+)} groups. NE, not estimable: The odds ratio could not be estimated due to zero cell count in the contingency table. ED: emergency department; GCS: Glasgow Coma Scale; RTS: Revised Trauma Score; AO/OTA: Association for Osteosynthesis Foundation and Orthopedic Trauma Association; AIS: Abbreviated Injury Scale; ISS: Injury Severity Score Statistical significance: *p < 0.05, **p < 0.01, ***p < 0.001

	ICU(-) group (n = 40)	ICU(+) group (n = 19)	Test statistic (Wilcoxon rank sum test, w/ odds ratio)	p-value
Age (y/o)	73 (55,79.25)	63 (36, 80.5)	455	0.227
Age > 60 y/o	29 (72.5%)	12 (63.2%)	0.655 (0.177-2.500)	0.550
Male	19 (47.5%)	11 (57.9%)	1.509 (0.442-5.344)	0.580
Ingestion of antithrombotic agents before	5 (12.5%)	2 (10.5%)	0.826 (0.072-5.713)	1.000
Injury mechanism: high-energy	25 (62.5%)	19 (100%)	NE	0.001**
Vital signs on arrival at our ED				
Heart rate	81.5 (71.0, 88.5)	103 (86, 113.0)	188	0.002**
Heart rate > 100	3 (7.5%)	10 (52.6%)	12.908 (2.644-88.235)	<0.001***
Systolic blood pressure	133 (122, 166)	112 (99, 138)	539	0.010*
Systolic blood pressure < 90	1 (2.5%)	3 (15.8%)	7.042 (0.522-392.450)	0.093
Respiratory rate	18 (17.75, 20.0)	23 (17.5, 27.5)	252	0.037*
Respiratory rate > 24	3 (7.5%)	6 (31.6%)	5.497 (1.004-38.981)	0.025*
GCS	15 (14.75, 15)	14 (14, 15)	499	0.021*
GCS < 15	10 (25.0%)	10 (52.6%)	3.258 (0.906-12.260)	0.045*
RTS < 7.0	0 (0%)	4 (21.1%)	NE	0.009**
Fracture types				
AO/OTA-type C	1 (2.5%)	2 (10.5%)	4.457 (0.218-276.926)	0.240
AO/OTA-type B	3 (7.5%)	4 (21.1%)	3.214 (0.481-24.670)	0.197
AO/OTA-type A	36 (90.0%)	13 (68.4%)	0.248 (0.043-1.232)	0.062
AO/OTA unstable	4 (10.0%)	6 (31.6%)	4.039 (0.811−22.814)	0.062
Ilium fracture	17 (42.5%)	9 (47.4%)	1.214 (0.351-4.181)	0.784
Pubis fracture	17 (42.5%)	16 (84.2%)	6.976 (1.627-43.313)	0.004**
Ischium fracture	21 (52.5%)	13 (68.4%)	1.938 (0.549−7.540)	0.275
Sacrum fracture	10 (25.0%)	7 (36.8%)	1.733 (0.448−6.536)	0.372
Acetabulum fracture	12 (30.0%)	6 (31.6%)	1.076 (0.269−4.000)	1.000
Ilium fracture + others	6 (15.0%)	6 (31.6%)	2.569 (0.573−11.654)	0.174
Pubis + others	16 (40.0%)	16 (84.2%)	7.715 (1.796−47.957)	0.002**
Ischium + others	18 (45.0%)	13 (68.4%)	2.604 (0.739−10.160)	0.105
Sacrum + others	7 (17.5%)	7 (36.8%)	2.698 (0.657−11.282)	0.117
Acetabulum + others	11 (27.5%)	6 (31.6%)	1.213 (0.300−4.583)	0.766
Unilateral fracture	20 (50.0%)	10 (52.6)	1.109 (0.325−3.833)	1.000
Bilateral fracture	0 (0%)	6 (31.6%)	NE	<0.001***
Single fracture	15 (37.5%)	1 (5.3%)	0.096 (0.002−0.732)	0.001*
Multiple fractures	25 (62.5%)	18 (94.7%)	10.471 (1.366−478.273)	0.001*
Right multiple fractures	11 (27.5%)	11 (57.9%)	3.538 (0.998−13.323)	0.042*
Left multiple fractures	9 (22.5%)	11 (57.9%)	4.594 (1.259−18.046)	0.017*
Bilateral multiple fractures	0 (0%)	6 (31.6%)	NE	<0.001***
Number of fractures	2 (1, 3)	4 (2, 4.5)	177	<0.001***
Fracture ≥ 2	21 (52.5%)	16 (84.2%)	4.705 (1.096−29.134)	0.023*
Fracture ≥ 3	12 (30.0%)	13 (68.4%)	4.903 (1.356−19.871)	0.010*
Fracture ≥ 4	5 (12.5%)	11 (57.9%)	9.152 (2.213−44.299)	<0.001***
Fracture ≥ 4 and ilium fracture	3 (7.5%)	4 (21.1%)	3.215 (0.481−24.670)	0.198
Fracture ≥ 4 and pubis fracture	5 (12.5%)	11 (57.9%)	9.152 (2.213−44.299)	<0.001***
Fracture ≥ 4 and ischium fracture	5 (12.5%)	10 (52.6%)	7.443 (1.796−35.589)	0.003**
Fracture ≥ 4 and sacrum fracture	3 (7.5%)	5 (26.3%)	4.278 (0.724−31.285)	0.097
Fracture ≥ 4 and acetabulum fracture	3 (7.5%)	5 (26.3%)	4.278 (0.724−31.285)	0.097
Pelvis AIS	2 (2, 2)	2 (2, 2)	357.5	0.599
ISS ≧ 16	3 (7.5%)	13 (68.4%)	24.464 (4.917−175.252)	<0.001***
Organ damage (including duplications)	8 (20.0%)	11 (57.9%)	5.314 (1.427−21.520)	0.007**
Brain	3 (7.5%)	5 (26.3%)	4.278 (0.724−31.285)	0.097
Lung	4 (10.0%)	9 (47.4%)	7.745 (1.732−42.157)	0.002**
Spleen	0 (0%)	2 (10.5%)	NE	0.100
Liver	0 (0%)	1 (5.3%)	NE	0.322
Kidney	0 (0%)	3 (15.8)	NE	0.030*
Extravasation in CT findings	2 (5.0%)	7 (36.8%)	10.543 (1.711−117.343)	0.003**
Endovascular treatment	0 (0%)	7 (36.8%)	NE	<0.001***
Blood infusion	0 (0%)	12 (63.2%)	NE	<0.001***
Massive transfusion	0 (0%)	4 (21.1%)	NE	0.009**
Death in our hospital	0 (0%)	1 (5.2%)	NE	0.322

**Figure 2:**
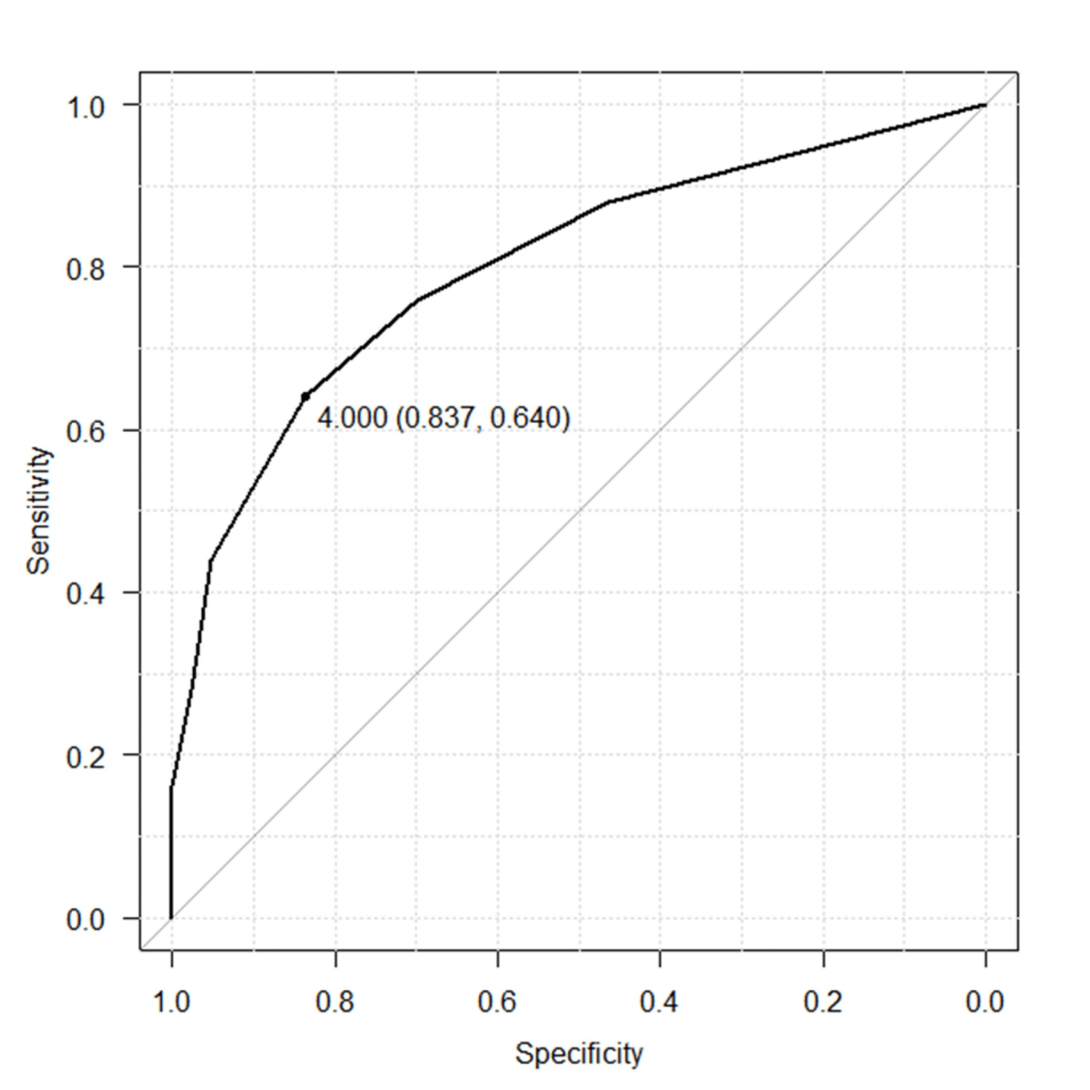
Area under receiver operating characteristic curves between the number of pelvic fractures and ICU admission. The number of pelvic fractures was evaluated using the receiver operating characteristic curve analysis as a predictor for ICU admission. The area under the curve (AUC) was 0.767 (95% confidence interval (CI), 0.631-0.903). For a cut-off point of 4.0 pelvic fractures, the maximum sensitivity was 83.7%, specificity was 64.0%, positive predictive value (PPV) was 49.7%, and negative predictive value (NPV) was 90.7%.

Significant differences were observed in the mechanism of injury, heart rate, presence of tachycardia, systolic blood pressure, respiratory rate, presence of tachypnea, Glasgow Coma Scale, and presence of disturbed consciousness. Regarding fracture type, there were no significant differences for unstable pelvic fractures (AO classification type B, C). However, there were significant differences for pubic fractures, combined pubic and other fractures, multiple fractures, bilateral fractures, unilateral multiple fractures, and the number of fractures. For the number of fractures, the receiver operating characteristic curve analysis revealed a threshold of four fractures for ICU admission (area under the curve 0.767, 95% CI 0.631-0.903) (Figure 2).

In addition, outcomes such as ISS ≥ 15, presence of organ injury (particularly lung injury), extravasation on contrast-enhanced CT, performance of endovascular treatment, and blood transfusion were significantly more common in ICU-admitted patients.

Further analysis of pubic fractures (Table 3) showed that although patients with pubic fractures had significantly lower systolic blood pressure on arrival, there was no significant difference in the presence of shock on arrival.

**Table 3:** Comparison of the groups with pubic fracture {P(+)} and without pubic fracture {P(-)}. NE, not estimable: The odds ratio could not be estimated due to zero cell count in the contingency table. GCS: Glasgow Coma Scale Statistical significance: * p < 0.05, ** p < 0.01, *** p < 0.001​

	P(+) n = 33	P(-) n = 26	Test statistic (Wilcoxon rank sum test, w/ odds ratio (95%CI))	P-value
Age	68 (48.0, 78)	73 (62.5, 80)	488.5	0.368
Male	18 (54.5%)	12 (46.2%)	1.392 (0.443−4.449)	0.604
Heart rate	89 (74.0, 105)	82.5 (71.25, 89)	310	0.070
Systolic blood pressure	128 (108.0, 142)	143 (116.75, 172)	580	0.022*
Shock	4 (12.1%)	0 (0.0%)	NE	0.123
Respiratory rate	19.0 (18, 23.0)	18.5 (17, 21.5)	384	0.494
GCS	15 (14.0, 15)	15 (14.25, 15)	493.5	0.242
Anticoagulant	4 (12.1%)	3 (11.5%)	1.056 (0.161−7.945)	1.000
High-energy	28 (84.8%)	16 (61.5%)	3.422 (0.879−15.174)	0.069
Multiple fractures	32 (97.0%)	11 (42.3%)	40.575 (5.181−1870.603)	<0.001***
Extravasation	9 (27.3%)	0 (0.0%)	NE	0.003**
Organ damage	5 (15.2%)	14 (53.8%)	3.036 (0.831−12.900)	0.092
Brain	6 (18.2%)	2 (7.7%)	2.626 (0.417−29.002)	0.446
Lung	10 (30.3%)	3 (11.5%)	3.269 (0.716−20.876)	0.117
Blood infusion	12 (36.4%)	0 (0.0%)	NE	<0.001***
Endovascular treatment	7 (21.2%)	0 (0.0%)	NE	0.015*
Dead	1 (3.0%)	0 (0.0%)	NE	1.000

Patients with pubic fractures frequently had multiple fractures and were significantly more likely to show contrast extravasation. While bleeding from associated fracture sites was observed, eight out of nine cases with extravasation exhibited bleeding near the pubic fracture site. These patients also frequently received blood transfusions.

A logistic regression analysis was conducted using specific fracture sites (ilium, pubis, ischium, sacrum, and acetabulum) as explanatory variables for ICU admission. Among these sites, the presence of a pubic fracture was identified as an independent risk factor (OR, 11.9; P = 0.0122). Furthermore, in a subsequent model adjusted for age, sex, and the presence of organ injury, the pubic fracture remained a significant independent risk factor for ICU admission, alongside organ injury (Table 4).

**Table 4:** Logistic regression analysis for ICU admission. This analysis performed multivariate logistic regression using ICU admission as the dependent variable across two different models. Regarding goodness-of-fit, Model 1 (AIC = 74.2) showed a significant improvement compared to the null model (deviance χ^2^ = 11.95, p = 0.035), and Model 2 (AIC = 68.9) showed a more highly significant improvement (deviance χ^2^ = 15.24, p = 0.004). Furthermore, the Variance Inflation Factor (VIF) calculated for all independent variables in each model was below 2.0 (maximum VIF of Model 1 = 1.97, maximum VIF of Model 2 = 1.07), indicating that there is no issue of multicollinearity. ICU: intensive care unit Statistical significance: * p < 0.05, ** p < 0.01

	Odds ratio	95% CI lower bound	95% CI upper bound	P-value
Model 1				
Intercept	0.0759	0.00145	0.398	0.002**
Ilium fracture	2.3400	0.5900	9.290	0.227
Pubis fracture	11.9000	1.7100	82.400	0.012*
Ischium fracture	0.6500	0.1130	3.740	0.629
Sacrum fracture	1.4200	0.3250	6.220	0.640
Acetabulum fracture	1.1100	0.2550	4.830	0.889
Model 2				
Intercept	0.077	0.012	0.482	0.006
Age >60 y/o	1.070	0.266	4.270	0.928
Sex (man)	1.110	0.309	4.020	0.868
Organ damage	4.370	1.170	16.300	0.029*
Pubis fracture	5.960	1.390	25.500	0.016*

## Discussion

Trauma is the leading cause of death for patients aged 15-24 years and accounts for approximately 30% of annual ICU admissions [18]. Among these, the mortality rate for pelvic fractures is reported to be 6-31% [19-21], and its mere presence has traditionally been considered to be a severity factor [6,22]. However, recent studies suggest that pelvic fractures themselves may have a low likelihood of severe outcomes, with the presence of concomitant organ damage or vascular injury being more critical [5,6]. Previous studies have identified factors such as pre-existing frailty [23], associated organ injury [19], and thoracolumbar spinal injury [24] as increasing the odds of ICU admission. However, to the best of our knowledge, no studies have discussed which specific pelvic fracture patterns, when considered in isolation, lead to ICU admission.

In this study, the most common cause of pelvic fracture was traffic accidents (30/59 cases). This aligns with multiple epidemiological studies indicating that traffic accidents are the most frequent cause [25-27]. The median patient age was 69 years (51.5, 79.5), and 19/59 patients (32.2%) were admitted to the ICU. The median AIS was 2. In two facilities in Qatar and Germany, 22% of pelvic fracture patients were admitted to the ICU [27]. A study focusing on patients aged 65 years or older with a pelvic AIS ≥ 3 reported an ICU admission rate of 46% [28]. Our study had a higher ICU admission rate, but the median patient age exceeded 65 years, placing our admission rate within the expected range of previous studies. Giannoudis et al. [19] conducted a study involving 11,149 patients with traumatic pelvic fractures and found that 21% of patients with pelvic fractures also had severe chest trauma with AIS ≥ 3, while 17% had head trauma. In this study, 13/59 (22%) patients had chest trauma with AIS ≥ 3, and 8/59 (13.6%) had head and neck trauma with AIS ≥ 3, which was similar to findings in earlier studies.

The mortality rate in our study was 1.7% (1/59). Mortality rates for pelvic fractures vary widely across reports [5,19,29,30]. At our institution, external fixation was performed in only one case, and internal fixation surgery was conducted in another case. Interventional radiology was performed in 7/59 cases. Moreover, 72.9% of pelvic fracture patients had multiple fractures. Consistent with previous reports, patients with organ damage were more likely to be admitted to the ICU.

Our analysis of pelvic fracture patients revealed that having multiple fractures and a higher number of fracture sites significantly increased ICU admission rates. Furthermore, the presence of a pubic fracture was associated with ICU admission (p = 0.00435). While patients with pubic fractures had significant differences in blood pressure on arrival, there was no difference in the incidence of shock. Patients with pubic fractures had multiple fractures and significantly more frequent extravasation. Although bleeding from associated fracture sites was also observed, eight out of nine cases with extravasation had bleeding near the pubic fracture site. Many patients received blood transfusions. The pubic region contains arteries such as the obturator artery and internal pudendal artery, which are susceptible to injury with pubic fractures [31,32].

While there was no difference in pelvic AIS for ICU-admitted pelvic fractures, these patients had significantly higher ISS (ISS ≥ 15), indicating a greater anatomical severity of overall trauma. The presence of a pubic fracture alone was suggested as an independent factor for ICU admission, even when compared to previously recognized organ injuries.

Classifications such as Young-Burgess and Tile [33] have reported some inter- and intra-observer inconsistencies [34-36]. Our findings, which suggest that specific fracture patterns can be predictors of ICU admission without relying on complex classifications like Tile or AO/OTA, may contribute to rapid decision-making in initial patient management.

Although further validation with a larger cohort is necessary, our institution observed the following trend. If a pubic fracture is identified as a factor for ICU admission, it may be advisable to transport patients with pubic symphysis tenderness in pre-hospital settings to medical facilities capable of ICU management, even in cases involving minor mechanisms of injury.

Furthermore, recent efforts in traffic accident prevention have increasingly utilized computer simulation systems instead of traditional simulator-based crash tests. THUMS, a prominent example, acquires bone tissue information from various autopsy cases and reproduces its fragility with relatively high accuracy. However, biological information on internal organs and blood vessels is not yet reflected, making accurate evaluation difficult. Continuing a series of bone-focused studies, like this present investigation, might contribute to vehicle development that could prevent cases requiring transport to high-level medical institutions.

Limitations

Since this was an observational study, causal inferences cannot be made. Despite controlling for several covariates, the presence of residual confounding (i.e., unmeasured or unknown confounders) is a limitation inherent to this type of observational design. This was also a single-center retrospective study rather than a prospective one. The small sample size of the analyzed cohort limits the statistical power and generalizability of the findings. The median age of the cohort exceeded 65 years, indicating a relatively older patient group. ICU admission decisions were based on the comprehensive judgment of the attending physician, which may introduce some subjectivity. The fracture cohort exhibited a low-severity profile, with patterns predominantly classified as AO/OTA Type A. This bias may limit the generalizability to more complex injuries. The study environment was unique due to limited surgical interventions, including external and internal fixation. Cases of pelvic fractures that were brought in by family or transferred from other hospitals due to severe conditions were excluded; thus, the findings may not be applicable to all pelvic trauma cases.

## Conclusions

Our study revealed that a pubic fracture is a significant independent risk factor for ICU admission in pelvic fracture patients, with an OR of 11.9. Furthermore, patients with multiple fracture sites or four or more fractures showed significantly higher ICU admission rates. This suggests that specific fracture patterns, such as a pubic fracture, can serve as predictors for ICU admission. These findings have the potential to aid in rapid decision-making during the initial assessment and management of patients in emergency settings.

## References

[1] Abrassart S, Stern R, Peter R (2013). Unstable pelvic ring injury with hemodynamic instability: what seems the best procedure choice and sequence in the initial management?. Orthop Traumatol Surg Res.

[2] Eastridge BJ, Starr A, Minei JP, O'Keefe GE, Scalea TM (2002). The importance of fracture pattern in guiding therapeutic decision-making in patients with hemorrhagic shock and pelvic ring disruptions. J Trauma.

[3] Balogh Z, Caldwell E, Heetveld M, D'Amours S, Schlaphoff G, Harris I, Sugrue M (2005). Institutional practice guidelines on management of pelvic fracture-related hemodynamic instability: do they make a difference?. J Trauma.

[4] Cothren CC, Osborn PM, Moore EE, Morgan SJ, Johnson JL, Smith WR (2007). Preperitonal pelvic packing for hemodynamically unstable pelvic fractures: a paradigm shift. J Trauma.

[5] Holstein JH, Culemann U, Pohlemann T (2012). What are predictors of mortality in patients with pelvic fractures?. Clin Orthop Relat Res.

[6] Sathy AK, Starr AJ, Smith WR, Elliott A, Agudelo J, Reinert CM, Minei JP (2009). The effect of pelvic fracture on mortality after trauma: an analysis of 63,000 trauma patients. J Bone Joint Surg Am.

[7] Holtenius J, Bakhshayesh P, Enocson A (2018). The pelvic fracture - indicator of injury severity or lethal fracture?. Injury.

[8] Baker SP, O'Neill B, Haddon W Jr, Long WB (1974). The injury severity score: a method for describing patients with multiple injuries and evaluating emergency care. J Trauma.

[9] Balogh ZJ, Varga E, Tomka J, Süveges G, Tóth L, Simonka JA (2003). The new injury severity score is a better predictor of extended hospitalization and intensive care unit admission than the injury severity score in patients with multiple orthopaedic injuries. J Orthop Trauma.

[10] Champion HR, Sacco WJ, Copes WS, Gann DS, Gennarelli TA, Flanagan ME (1989). A revision of the Trauma Score. J Trauma.

[11] Le Gall JR, Lemeshow S, Saulnier F (1993). A new Simplified Acute Physiology Score (SAPS II) based on a European/North American multicenter study. JAMA.

[12] Kahloul M, Bouida W, Boubaker H (2014). Value of anatomic and physiologic scoring systems in outcome prediction of trauma patients. Eur J Emerg Med.

[13] Lavoie A, Moore L, LeSage N, Liberman M, Sampalis JS (2005). The Injury Severity Score or the New Injury Severity Score for predicting intensive care unit admission and hospital length of stay?. Injury.

[14] Rio TG, Nogueira LS, Lima FR, Cassiano C, Garcia DF (2023). Performance of severity indices for admission and mortality of trauma patients in the intensive care unit: a retrospective cohort study. Eur J Med Res.

[15] Association for the Advancement of Automotive Medicine (2008). Abbreviated Injury Scale (AIS) 2005 Update 2008. The Association for the Advancement of Automotive Medicine.

[16] Baidwan NK, Schauer SG, Dixon JM (2023). Tranexamic acid improves survival in the setting of severe head injury in combat casualties. Med J (Ft Sam Houst Tex).

[17] Niedermeier SR, Khan SN (2017). Polytrauma patients with associated spine fractures: an assessment of surgical intervention on patient outcome. Clin Spine Surg.

[18] Mackenzie EJ, Rivara FP, Jurkovich GJ (2007). The national study on costs and outcomes of trauma. J Trauma.

[19] Giannoudis PV, Grotz MR, Tzioupis C, Dinopoulos H, Wells GE, Bouamra O, Lecky F (2007). Prevalence of pelvic fractures, associated injuries, and mortality: the United Kingdom perspective. J Trauma.

[20] Palmcrantz J, Hardcastle TC, Naidoo SR, Muckart DJ, Ahlm K, Eriksson A (2012). Pelvic fractures at a new level 1 trauma centre: who dies from pelvic trauma? The Inkosi Albert Luthuli Central Hospital experience. Orthop Surg.

[21] Demetriades D, Karaiskakis M, Toutouzas K (2002). Pelvic fractures: epidemiology and predictors of associated abdominal injuries and outcomes. J Am Coll Surg.

[22] Chong KH, DeCoster T, Osler T, Robinson B (1997). Pelvic fractures and mortality. Iowa Orthop J.

[23] Forssten MP, Sarani B, Mohammad Ismail A, Cao Y, Ribeiro MA Jr, Hildebrand F, Mohseni S (2023). Adverse outcomes following pelvic fracture: the critical role of frailty. Eur J Trauma Emerg Surg.

[24] Mohs ZA, Albrecht N, Duncan AJ, Cao L, Ahmeti M (2025). Pelvic fractures and thoracolumbar spine injury: a critical overlook in high-impact vehicular trauma management. Injury.

[25] Hossain A, Islam S, Haque Qasem MF, Faisal Eskander SM, Hasan MT, Nahar M (2020). Epidemiology of pelvic fractures in adult: our experience at two tertiary care hospital in Dhaka, Bangladesh. J Clin Orthop Trauma.

[26] Ghosh S, Aggarwal S, Kumar V, Patel S, Kumar P (2019). Epidemiology of pelvic fractures in adults: our experience at a tertiary hospital. Chin J Traumatol.

[27] Abdelrahman H, El-Menyar A, Keil H (2020). Patterns, management, and outcomes of traumatic pelvic fracture: insights from a multicenter study. J Orthop Surg Res.

[28] Garcia M, Firek M, Zakhary B, Brenner M, Hildebrand F, Coimbra R (2020). Severe pelvic fracture in the elderly: high morbidity, mortality, and resource utilization. Am Surg.

[29] Papakostidis C, Giannoudis PV (2009). Pelvic ring injuries with haemodynamic instability: efficacy of pelvic packing, a systematic review. Injury.

[30] Grotz MR, Allami MK, Harwood P, Pape HC, Krettek C, Giannoudis PV (2005). Open pelvic fractures: epidemiology, current concepts of management and outcome. Injury.

[31] Kachlik D, Vobornik T, Dzupa V (2019). Where and what arteries are most likely injured with pelvic fractures?: The influence of localization, shape, and fracture dislocation on the arterial injury during pelvic fractures. Clin Anat.

[32] Heichinger R, Pretterklieber ML, Hammer N, Pretterklieber B (2023). The Corona mortis is similar in size to the regular obturator artery, but is highly variable at the level of origin: an anatomical study. Anat Sci Int.

[33] Zingg T, Uldry E, Omoumi P (2021). Interobserver reliability of the Tile classification system for pelvic fractures among radiologists and surgeons. Eur Radiol.

[34] Tile M (1996). Acute pelvic fractures: I. Causation and classification. J Am Acad Orthop Surg.

[35] Koo H, Leveridge M, Thompson C (2008). Interobserver reliability of the young-burgess and tile classification systems for fractures of the pelvic ring. J Orthop Trauma.

[36] Furey AJ, O'Toole RV, Nascone JW, Sciadini MF, Copeland CE, Turen C (2009). Classification of pelvic fractures: analysis of inter- and intraobserver variability using the Young-Burgess and Tile classification systems. Orthopedics.

